# Application of Alginate Hydrogels for Next-Generation Articular Cartilage Regeneration

**DOI:** 10.3390/ijms23031147

**Published:** 2022-01-20

**Authors:** Wei Liu, Henning Madry, Magali Cucchiarini

**Affiliations:** Center of Experimental Orthopaedics, Saarland University Medical Center, Kirrbergerstr. Bldg 37, D-66421 Homburg (Saar), Germany; dr.wei.liu@hotmail.com (W.L.); henning.madry@uks.eu (H.M.)

**Keywords:** alginate, hydrogel, cell therapy, gene therapy, scaffold-guided gene transfer, cartilage regeneration

## Abstract

The articular cartilage has insufficient intrinsic healing abilities, and articular cartilage injuries often progress to osteoarthritis. Alginate-based scaffolds are attractive biomaterials for cartilage repair and regeneration, allowing for the delivery of cells and therapeutic drugs and gene sequences. In light of the heterogeneity of findings reporting the benefits of using alginate for cartilage regeneration, a better understanding of alginate-based systems is needed in order to improve the approaches aiming to enhance cartilage regeneration with this compound. This review provides an in-depth evaluation of the literature, focusing on the manipulation of alginate as a tool to support the processes involved in cartilage healing in order to demonstrate how such a material, used as a direct compound or combined with cell and gene therapy and with scaffold-guided gene transfer procedures, may assist cartilage regeneration in an optimal manner for future applications in patients.

## 1. Introduction

The articular cartilage, the gliding avascular tissue covering the extremities of articulating bones in synovial joints, is a specialized tissue essentially composed of chondrocytes, producing a dense extracellular matrix (ECM) in a highly defined territorial matrix microstructure and zonal cartilage macrostructure. Articular cartilage lesions that occur during trauma, with the degradation of the ECM together with chondrocyte alterations, are common clinical problems that remain challenging, as none of the current conservative and surgical treatments reliably restore the original hyaline cartilage tissue with complete structural and functional integrity in sites of injury [1]. Reparative processes are also impeded by the improper intrinsic ability of the articular cartilage to self-heal, with its potentially unstable chondrocyte phenotype [2,3], showing the urgent need for more effective therapeutic options capable of enhancing the processes of cartilage regeneration.

**Figure 1:**
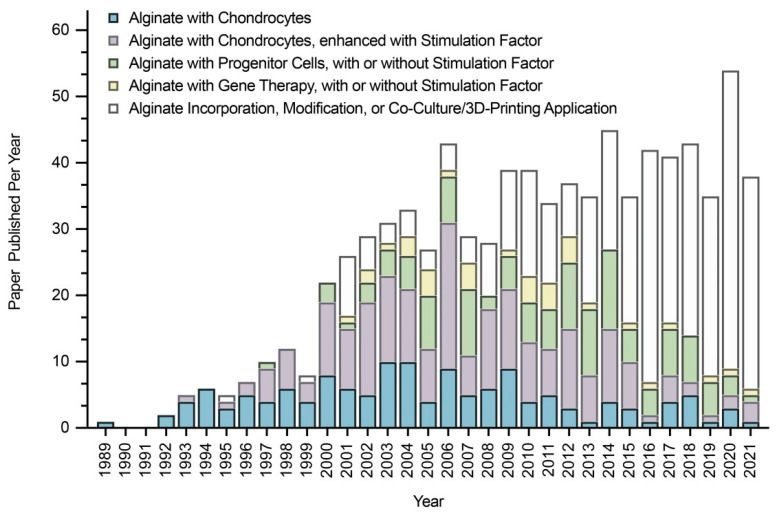
Research progress on alginate for cartilage regeneration (created with Prism).

In this regard, alginate, a compound based on a polysaccharide from brown algae, displays several advantageous features that make it a particularly well-suited material in tissue engineering approaches for cartilage regeneration. Alginate is a biocompatible component with a high water content and good porosity and with tunable viscosity, broadly reported for its ability to easily form hydrogels that can be used as scaffolds to load cells and drugs [4]. Extensive work has described using alginate-based hydrogels as therapeutic platforms for applications in cartilage regeneration, with such systems based on continuous progress and advances, leading to emerging systems that can be adapted to fit sites of cartilage injury using reparative cells, external stimuli, and a variety of modifications of the alginate systems (Figure 1).

The goal of this work is to present a comprehensive appraisal of the potential applications of alginate for cartilage regeneration, covering experimental and preclinical data, in vitro and in vivo, and the currently available clinical protocols. A further goal is to discuss the benefits of each approach developed, especially those based on the incremental use of chondro-reparative cells (chondrocytes, progenitor cells) and biological/physicochemical stimuli (recombinant factors, mechanical compression, oxygen tension, osmotic pressure, ultrasounds), as well as their specific limitations. Emerging systems to tackle these limitations are also introduced and discussed, with a special focus on using alginate in combination with gene therapy and with other compounds or modifications that may altogether lead to next-generation, better-adapted alginate systems for effective translational applications in cartilage regeneration to treat patients in the future.

## 2. Articular Cartilage: Functions, Structure, Injuries, and Current Options

### 2.1. Functions and Structure of Articular Cartilage

The articular cartilage is the gliding tissue in the joints [5] that supports load transmission and translation, shock absorption, and friction reduction in synovial articulations [6,7]. The biological structure of the articular cartilage is critical to its functions [8].

#### 2.1.1. Function and Microstructure

The articular cartilage is composed of chondrocytes that produce and surround themselves with a highly hydrated ECM [5,9,10]. The chondrocytes represent the main cell type in the articular cartilage that also includes a population of stem cells [11] or progenitor cells [12,13,14]. Altogether, these cells contribute to only 5% of the total cartilage volume [5]. At the adult age, these cells rarely divide, but they maintain an ability to produce and retain this ECM after skeletal maturity [15]. The ECM is composed of a pericellular, territorial, and interterritorial matrix [16,17]. The extensively studied territorial matrix can be considered to be a chondron-surrounding structure consisting of a high concentration of soluble proteoglycans (PGs) with rapid turnover, embedded in a dense meshwork of fibrous proteins with low turnover [5,18,19,20,21]. PGs used as a cell cushion consist of a protein core surrounded by long chains of starch-like molecules called glycosaminoglycans (GAGs) [22] which can be classified as large, predominant PGs, such as aggrecan, and as small, minor PGs, such as decorin, biglycan, asporin, lumican, and fibromodulin [18,23,24]. GAGs include hyaluronic acid (HA), dermatan sulfate, chondroitin sulfate, heparan sulfate, and keratan sulfate [22], while the major GAGs attached to the core protein include chondroitin-4/6-sulfate and keratan sulfate [22]. Additionally, non-HA GAGs interacting with HA leads to PGs forming large, multi-molecular aggregates (aggregating PGs) [25]. Aggrecan, also known as the cartilage-specific proteoglycan core protein (CSPCP), or chondroitin-1-sulfate, is a protein encoded by the aggrecan gene in humans [26], and always represents the predominant population of the PGs synthesized [22,27]. Its size varies depending on the age of the cartilage from which the cells are derived [27]. Small PGs are represented by decorin and biglycan, with leucine-rich regions in their core protein and chondroitin sulfate or dermatan sulfate chains, respectively [28], and interact with type-II collagen [18], taking 1–2% of the total mass of the PGs as essential components of the normal mature articular cartilage [29]. The fibrous proteins used to support cells are composed of insoluble structural proteins, providing strength and resilience (type-II collagen and other collagens) and elastin and soluble specialized proteins that bind PGs and collagen fibers to receptors on the cell surface (fibronectin, laminin) [5,18,20,21]. Type-II collagen is the basis for the hyaline cartilage formed by the homotrimers of type-II collagen alpha 1 chains, representing up to 50% of all proteins and 85–90% of collagens in the tissue [20,23,28,30,31,32,33,34,35,36,37]. Other collagens include smaller amounts of type-VI [23,30,31,32,33,37], -IX [20,23,28,30,31,32,33,34,35,36,37], -XI [20,23,28,30,31,32,33,34,35,36,37], and -XIV collagen [30,31,32,37], and some type-I/-X collagen [38], which play important roles in the formation and stability of fibrils in the mature articular cartilage [39]. Compared with the territorial matrix, a variety of matrix molecules, such as aggrecan monomers and small aggregates [40,41], type-VI [42,43] and -IX collagen [44,45], hyaluronan [41,46], biglycan [47], and perlecan [48], can be found either exclusively or at higher concentration in the pericellular matrix. Interactions between the molecular constituents of the ECM contribute to the distinct macrostructure of the ECM. Large quantities of water are confined by PGs, contributing to up to 70–80% of the cartilage wet weight [31], opposing the deformation caused by compressive loading and tensile loading in the joint.

#### 2.1.2. Function and Macrostructure

The articular cartilage is subdivided into the superficial, middle, and deep zones [5,9,10]. The superficial, tangential zone is composed of ellipsoidal cells aligned parallel to the surface [5]. In this zone, the chondrocytes have the smallest size [15] and the cell density is the most elevated [15,49,50,51]. The superficial zone represents approximately 10% of the articular cartilage volume [52], with the lowest biosynthetic activity [51] and the highest amounts of small PG [53,54] and type-II [55] and -I collagen [56] deposition. The deposition of small PGs and type-II collagen decreases with the increase in the distance from the superficial zone [38], while clusterin [57,58] and the superficial zone protein (SZP) [59,60,61] are found exclusively in this zone. Collagen fibers are orientated at high densities in bundles parallel to the articulating surface, along with the cells, giving the superficial zone the highest ability of tensile stiffness and strength [62,63]. The middle zone is composed of randomly distributed spherical chondrocytes within a matrix, with collagen fibrils arranged in an oblique orientation to the surface [5], displaying a middle size at the lowest cell density [15]. This zone approximately accounts for 60–70% of the articular cartilage volume [52], with a high deposition of HA [64], dermatan sulfate [64], and cartilage intermediate layer protein [65]. The deep zone contains the largest cells [15], which are arranged in columns aligned perpendicular to the surface [5] with a middle-range cell density [15]. This zone almost occupies 10–15% of the articular cartilage volume [52], with the highest amounts of large PG [55], type-X collagen [55], cartilage oligomeric protein (COMP) [66], and keratan sulfate [64,67] deposition. Chondroitin sulfate has been reported to have either the highest deposition in the deep [64] or in the middle zone [67], depending on the methods of evaluation and on the variability of the samples tested. Large PGs are orientated at a high density in vertical columns perpendicular to the articulating surface along with the cells, allowing the deep zone to have a higher ability of compressive and resilience [15]. Differences in cell morphology, cell densities, cell metabolism, and matrix biochemical composition in each zone are the reasons of the different functions in the zonal articular cartilage [24]. The assembly of biomolecules determines the functionally defined cartilage, supporting its ability to transmit load, absorb shock, and reduce friction [19], while alterations of any of its components may decrease its ability to withstand loads placed across it [8].

#### 2.1.3. Special Features

The special biological features of the articular cartilage include its low cellularity, low proliferation, low cell migration, low vascularization, and low nutrition and waste diffusion, which together explain why the articular cartilage is intrinsically unable to readily regenerate itself [68,69,70,71,72,73,74,75,76,77,78]. As a result, chondrocytes are difficult to obtain in large quantities, for instance, in implantation protocols in cartilage regeneration [27,36,79,80,81]. In particular, the culture and expansion of chondrocytes in vitro remains problematic [72,82], since these cells differentiate into different phenotypes during expansion culture [83,84], including via transdifferentiation (hypertrophy/osteoblast expression) [85,86] and dedifferentiation (fibroblast expression) [87,88] relative to the normal differentiation state (original expression). Normally differentiated chondrocytes are found at the early stages of the monolayer culture [89], with a main deposition of type-II collagen [90,91], aggregating PGs [92], and a zonal characteristic protein (such as SZP) [93], accompanied by low levels of alkaline phosphatase activity [83]. Transdifferentiation chondrocytes are detected in high-density monolayer or organoid cultures [89], with a main deposition of type-X collagen [35,83,94], non-aggregating PGs [25,83], and osteopontin [35,83,94], accompanied by high levels of alkaline phosphatase activity [83,95], matrix vesicle formation [83], and endochondral ossification [94], mostly occurring at a fetal stage [94]. Dedifferentiating chondrocytes are found in low-density monolayer cultures (majority) [35,96,97], with a main deposition of type-I/-III/-V collagen [21,34,98] and of non-aggregating PGs [25,89], accompanied by an increase in CD44 fragmentation [99] and a decrease in the expression of type-II transforming growth factor beta (TGF-β) receptor (TβRII) and in the TGF-β response [100], mostly occurring at an adult stage [33,36,94]. The chondrocyte phenotype is affected by factors such as the cell adhesion status and the cell shape, and it differs in monolayer and three-dimensional (3D) culture systems [25], indicating that geometric entrapment is essential for the maintenance of the chondrocyte phenotype in vitro [101]. Suspension with gel seems to be an excellent method of geometric embedment for chondrocytes [80,83], likely promoting the retention of the original phenotype [102,103] that is required for articular cartilage regeneration.

### 2.2. Articular Cartilage Injuries

Articular cartilage injuries resulting from trauma or other causes affect more than 60% of examined patients [104], and may further deteriorate if left untreated [105,106]. From a macroscopic perspective (morphology), changes affecting the articular cartilage include extended matrix discontinuities, clefts, and erosions, while from a microstructural point of view (pathology), they include chondrocyte hypertrophy, apoptosis and necrosis, and a loss of PGs [5], which gradually and eventually leads to the formation of large cartilage defects, classified as either chondral defects restricted to the articular cartilage or osteochondral defects that further disrupt the underlying subchondral bone [107,108]. The injured articular cartilage has a limited intrinsic capacity for self-healing and the chondrocytes adjacent to the lesions do not substantially contribute to the reconstruction of the cartilage [108].

### 2.3. Current Clinical Options to Enhance Articular Cartilage Regeneration

Clinical strategies to manage articular cartilage injuries include conservative treatments to reduce pain and/or maintain mobility and surgical treatments to replace the damaged (osteo)chondral tissue and/or to induce repair [108]. Conservative treatments include non-pharmacological interventions (physical therapy) and pharmacological treatments (oral, topical, and intra-articular applications), both of which have had mixed success [5,108]. Surgical interventions include marrow stimulation (microfracture), autologous chondrocyte implantation (ACI), and the transplantation of autologous or allogeneic osteochondral grafts [108]. Microfracture is an adapted procedure to repair small (but not large) defects, mostly in young patients (<40) and especially to treat femoral condyles rather than other parts of the knee joint [109]. ACI avoids immune rejection, but needs intensive surgery to acquire chondrocytes [109]. Osteochondral graft transplantation, using non-weight-bearing articular surfaces from the patient, is relatively simple to perform, and also avoids immune rejection, but it is also best-suited for small defects [109]. However, none of these options lead to full cartilage regeneration in the sites of lesions. Microfracture may lead to the development of a fibrocartilaginous repair tissue that lacks the original hyaline structure of the native cartilage and which is unable to withstand mechanical loading [30,37,108,110,111]. ACI may be complicated by phenotype changes to the chondrocytes (hypertrophy), insufficient regenerative cartilage, and disturbed fusion [30,37,108,110,111]. Else, osteochondral graft transplantation may be impeded by disease transmission, graft failure, donor site morbidity, donor-to-recipient site incongruity, and hemarthrosis [30,37,108,110,111]. Overall, such outcomes show the critical need for improved strategies for cartilage regeneration. In this regard, the use of alginate hydrogels may represent an adapted platform for therapy, as they are based on a biocompatible, easy-to-handle material that can accommodate cells (and even tissues), biological stimuli (recombinant factors and even genes), and which are tunable to fit in all types of cartilage lesions (regardless of their size and location and of the patient’s age). They are being approved for use in trials, as described in detail in the following sections.

## 3. Alginate: Characteristics and Properties for Hydrogel Preparation

### 3.1. Basic Knowledge

**Figure 2:**
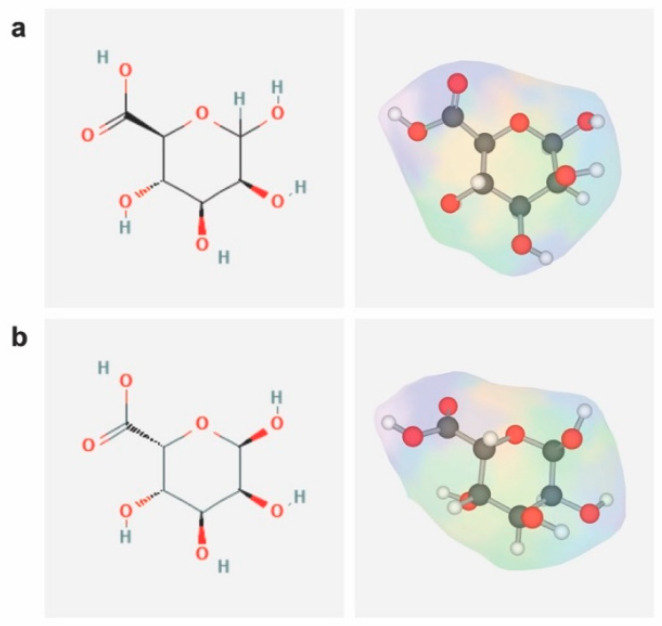
Structure of 1,4-linked β-D-mannuronic acid and 1,4 linked α-L-guluronic acid. The two-dimensional structures of (**a**) 1,4-linked β-D-mannuronic acid (CID: 439630) and (**b**) 1,4 linked α-L-guluronic acid (CID: 446401) were obtained from PubChem and their three-dimensional structures were drawn with LiteMol Viewer (1.6.5).

Alginates are unbranched linear copolymers composed of 1,4-linked β-D-mannuronic acid (M) and 1,4-linked α-L-guluronic acid (G), with regions exclusively composed of one unit or of the other (consecutive M block/residues, consecutive G block/residues), or with regions where the monomers approximate an alternating sequence (alternating MG block/residues) (Figure 2) [112,113].

Typically, alginate can be isolated from brown algae [114,115], which belong to Phaeophyceae (seaweeds) [112,116] or are extracted from bacteria, such as Pseudomonas or Azotobacter [117]. Alginate can be of nonbiomedical- or biomedical-grade quality (most employed) [103], of low (commonly used), medium, or high viscosity [103], and of low or high molecular weight (also termed polymer chain length) [112]. Alginate is hydrophilic, water soluble, and thickening in neutral conditions, and can form gel (hydrogel) when present in polyvalent cations [118,119].

### 3.2. Physicochemical Properties

#### 3.2.1. Gel Formation

Gelation occurs when polyvalent cations cooperatively interact with blocks of M/G monomers to form ionic bridges [112,117,120], followed by the formation of the 3D gel network via Van der Waals forces between alginate segments [121,122]. Cations that crosslink with alginate hydrogels include divalent cations (Ca^2+^, Ba^2+^, Sr^2+^, Cd^2+^, Co^2+^, Cu^2+^, Mn^2+^, Ni^2+^, Pb^2+^, and Zn^2+^) [82,117,123,124,125,126,127,128,129,130], trivalent cations (La^3+^, Pr^3+^, Nd^3+^, Eu^3+^, and Tb^3+^) [124], or multiple cations (Co^2+^ and Ca^2+^) [131], but neither Mg^2+^ nor monovalent cations [123,124,132]. During crosslinking, divalent metal ions exhibit a preference for GG blocks and lead to an increase in the extent of binding when increasing the ionic radius, while trivalent metal ions display a preference for GG blocks and lead to an increase in the extent of binding for both GG and MM blocks when increasing the charge density [124]. The contrast in the modes of interaction of divalent and trivalent cations with alginic acid may be related to differences in the coordination number and/or to hydration remaining in the inner sphere of ions [124].

The physicochemical and bioscaffolding properties of alginate have been reported to depend on the M/G ratio [133,134] and on the concentration of the alginate and gelling solutions (Ca^2+^) [120].

First, M/G ratios differ according to the source of raw material used in alginate manufacture [133,134], with alginates with a high M/G ratio primarily derived from Macrocystis pyrifera [112]/Durvillea potarum [135] and alginates with a low M/G ratio from Laminaria hyperborean [112]. M/G ratios also have an impact on the biological activities of the chondrocytes at encapsulation, including cell adhesion, colonization, migration, nutrition diffusion, and proliferation [125]. Alginates with a low M/G ratio retain greater tensile strength [125,134], offering a substrate against which traction can be exerted and, therefore, which aids cell adhesion, colonization, and subsequent migration [125]. Additionally, the diffusion of large molecules is impeded by alginate, an effect that is less evident for alginates with a low M/G ratio [136]. In this regard, alginates with a low M/G ratio allow for optimal cell proliferation [103]. The mechanical properties, including dynamic modulus, peak strain, and peak stress, are improved when using alginates with a low M/G ratio, while alginates with a high M/G ratio yield weaker and more elastic gels [112,129,137].

Second, alginate concentrations of 0.5–4% (*w*/*v*) and Ca^2+^ concentrations of 15–144 mM have been reported to be suitable for cartilage regeneration [138,139]. A gel with optimal handling characteristics can be obtained with alginate at 1–1.5% (*w*/*v*) and Ca^2+^ at 30–50 mM [120]. Most research was developed with alginate at 1.2% (*w*/*v*) and Ca^2+^ at 102 mM [68,140,141,142,143,144,145,146,147,148,149]. The adhesion, colonization, and migration of embedded cells are enhanced when increasing the concentration of alginate [136,150] or of Ca^2+^ [122,139], while the diffusion of large molecules becomes gradually impeded [136,151]. The effects of the alginate and Ca^2+^ concentrations occur in a dose-dependent manner [150,152]. Of note, the stiffness [136] and compressive modulus [150] increase with the increasing alginate concentration, while the shear moduli increase with higher Ca^2+^ concentrations [139]. At low Ca^2+^ concentrations, temporary gels can be obtained as highly viscose solutions, while stable gelation may result from permanent associations of crosslinking structures at high Ca^2+^ levels [122]. Specially, Ca^2+^ concentrations of 36, 72, and 144 mM, using alginate at 2%, has no effect on gel degradation [139].

#### 3.2.2. Gel Dissolution

Alginate gels dissolve upon the loss of polyvalent cations, releasing high and low molecular weight alginate strands [153] via spontaneous or induced dissolution.

Spontaneous dissolution occurs upon the substitution of crosslinking polyvalent ions with monovalent ions, such as Na^+^ [154]. Large differences are reported in dissolving times [155,156,157], possibly as a result of differences in experimental models, implant volumes, material forms, and external environments [125].

Induced dissolution occurs via the chelation of crosslinking polyvalent ions with a dissolving buffer [39,89,112,158], including ethylenediaminetetraacetic acid (EDTA) (50 mM) [81,158], citrate (55 mM sodium citrate in 0.15 M NaCl) [97,158], and phosphate solution [83,89]. Typically, 50 mM EDTA is more cytotoxic than 55 mM citrate, and 55 mM citrate is more cytotoxic than phosphate-buffered saline (PBS) when dissolved for the same period of time [159]. One of the major advantages of alginate is that the gel where the cells are entrapped can be dissolved with chelation [23,27].

#### 3.2.3. Gel Characteristics

Alginate gels can be prepared in a microscopically homogeneous manner [160], with a compression modulus from 1 to 1000 kPa [161] and a shear modulus from 0.02 to 40 kPa [161]. Such gels exhibit pH-responsive properties, with higher swelling ratios when increasing the pH values due to the chain expansion from the presence of ionic carboxylate groups on the backbone [117].

#### 3.2.4. Alginate Degradation

The shortcomings of alginate include a lack of biodegradability [153,162] due to its very slow degradation rate and an uncontrollable degradation pattern, resulting in the release of high molecular weight strands that may be difficult to clear from the body [117,119,153].

A partially oxidized alginate (periodate oxidation) may promote its hydrolysis (9 ds in PBS solution) with the cleavage of carbon–carbon bonds of cis-diol groups in uronate residues and an alteration of the chain conformation when crosslinking with calcium ions [153].

#### 3.2.5. Other Features

As a result of negative charges in alginate, the extracellular environment where cells may be maintained mirrors the cartilage ECM more closely than any other culture system [27].

As a natural polysaccharide, alginate exhibits a pH-dependent anionic nature and an ability to interact with cationic polyelectrolytes and PGs [117], making it a convenient system to entrap cells for in vitro growth [22,83].

In addition, while alginate may have different endotoxin contents depending on its level of purity, its relative stability with regard to biocompatibility has been demonstrated, supporting its adapted use as a biomaterial [103,163].

## 4. Alginate and Chondrocytes for Articular Cartilage Regeneration

Chondrocytes have been originally manipulated as a natural source of cells for entrapment in alginate for applications that aim to enhance the processes of cartilage regeneration.

### 4.1. Gelation

**Figure 3:**
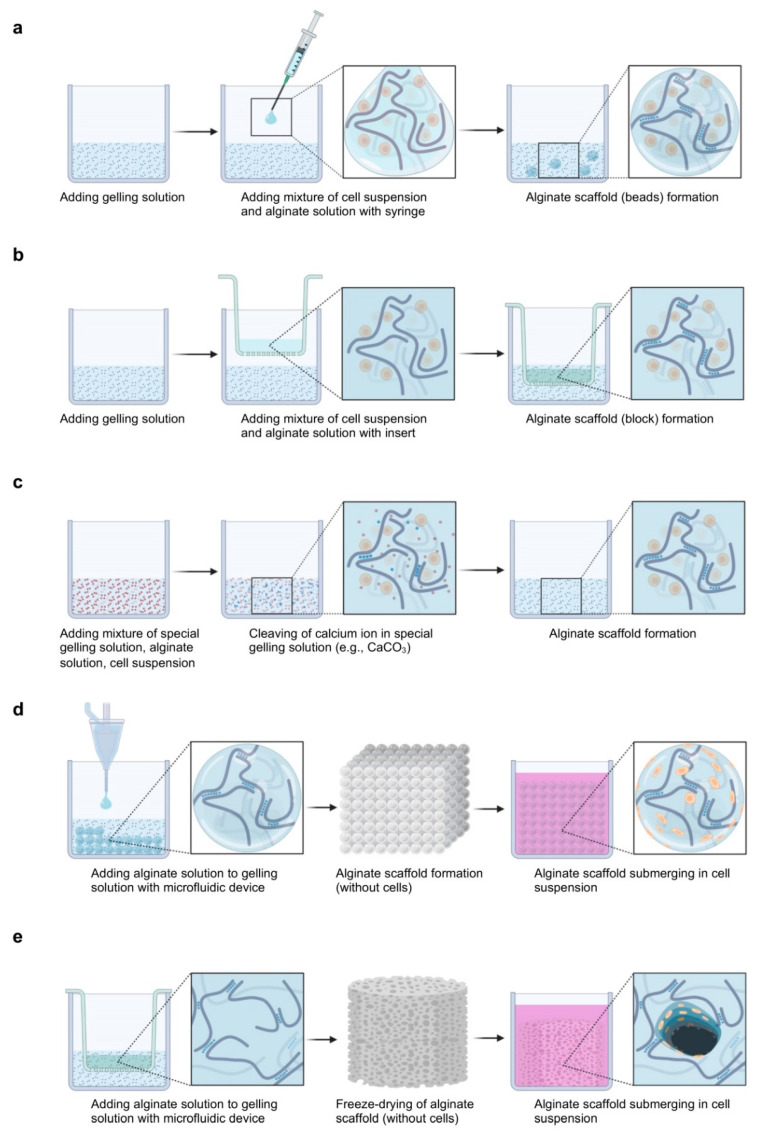
Fabrication of a cell–alginate hydrogel. (**a**–**c**) Cell encapsulation, (**d**,**e**) cell adhesion, (**a**,**b**,**d**,**e**) cation diffusion, and (**c**) cation dissociation (created with BioRender.com).

The process of alginate gelation with chondrocytes can be classified under cell encapsulation/adhesion or under cation diffusion/dissociation (Figure 3).

In the cell encapsulation and cation diffusion approach, the cell suspension is mixed with the alginate and gelling solutions using a syringe (this is the most used technique) [140,144], tips, or a Transwell insert [138,164,165].

In the cell encapsulation and cation dissociation approach, the cell suspension and alginate solution are mixed with a special gelling solution (e.g., CaCO_3_) where ions are gradually ionized [139].

In the cell adhesion and cation diffusion approach, the alginate and gelling solutions are mixed using microfluidics [166,167,168] or freeze-dried [117,169], followed by the seeding of the cell suspension.

The diffusion system is always associated with non-uniform gelation. The preparation of an alginate gel is a rapid process that limits the diffusion of gelling ions into the gel network so the heterogeneity can be reduced when a microstructure is used [170]. The rapid gelling process also limits the diffusion of gelling ions at different distances; thus, the heterogeneity can also be decreased when the sphere structure is employed [170]. Therefore, microbeads are mostly used in the literature [68,140,141]. Additionally, the diffusion system is usually associated with microchannel formation. Microchannels are formed by blocking the rapid diffusion of ions from the gelling solution into the boundary of the alginate solution and extending inward from the cells at the surface of the gel [160]. Microchannels are numerous when both the alginate and the gelling solutions lack Na^+^ ions or other monovalent cations [160]. Therefore, alginate is usually dissolved in NaCl (0.15 M) [145,171,172]. Furthermore, the dissociation system is often associated with ion-supersaturated gelation or semi-gelation. Alginate gels based on internal gelling have a more defined and limited supply of gelling ions compared with diffusion systems where ions are allowed to diffuse in the alginate solution to produce an ion-saturated gel [173]. In the presence of excessive amounts of gelling ions, the cell viability decreases, as gelling ions are cytotoxic before crosslinking and, conversely, semi-gelation occurs well with uncertain physiochemical and biological properties [159], making it difficult overall to obtain suitable conditions.

Key points to consider at gelation include the source of cells, the cell seeding density, and the environment and supporting medium. The chondrocytes used in cartilage research are various and include human [97,140] or non-human [140,167] cells, articular [171,174] or non-articular [95,175] chondrocytes, normal [27,174] or OA [176,177] cells, and superficial, middle, or deep zone [38] chondrocytes.

The diversity of the cell source employed may lead to different biomechanical and physiological results when applied for cartilage regeneration purposes. At various cell seeding densities, the ability of cell proliferation and/or of ECM deposition may also be affected [112], with a variability in the PG or collagen deposition [164,178] when modifying (increasing) the cell density. When increasing the cell seeding density at a prolonged culture time, the mechanical property (equilibrium tensile modulus) of the alginate gel increases [164] and the alginate beads have a tendency to crack and a great propensity to break apart [112].

Regarding the environment, decreases in the concentration of oxygen and of other nutrients in the center of the alginate impact the metabolism (reduction). Consequently, the rate of accumulation of ECM is limited by the rate of ECM production per cell and by the cell density that can be maintained in a viable state [178]. The administration of fetal bovine serum (FBS) results in the increased synthesis of ECM (PGs) per cell, but not in increased proliferation rates compared with serum-free conditions at short-term periods (equine and porcine chondrocytes) [112]. The application of human serum results in the increased deposition of ECM (PGs and type-II collagen) per cell but also in increased proliferation rates compared with FBS controls (human nasal septal chondrocytes from a young donor) [179].

Most studies have been performed using a Ca^2+^ concentration at 1.8 mM in culture medium after gelation, while the Ca^2+^ levels in the synovial fluid of normal human knee joints are 4 mM or even higher [158]. With a high-level concentration of Ca^2+^ (4–8 mM), the dynamic shear modulus, equilibrium shear modulus, and phase shift angle increase, but neither the equilibrium compressive modulus nor the total ECM (PGs and collagen) deposition increases [158].

Furthermore, a four-fold increase in glucose concentrations enhances the final mass of alginate by 10% and the deposition of PGs by 73% (5.1–20.4 mM), while a four-fold increase in the medium volume enhances the final mass of alginate by 44% and the deposition of PGs by 207% (0.4–1.6 mL/10^−6^ cells) [180].

### 4.2. In Vitro Studies

#### 4.2.1. Cell Morphology

Chondrocytes from different sources maintain a characteristically differentiated morphology in alginate throughout in vitro culture conditions [22,23,34,83,114]. After one day, the cells develop a spherical appearance and are irregularly scattered in the gels [83].

The cell cytoplasm contains a scarce amount of cisternae of the granular endoplasmic reticulum, few mitochondria, free ribosomes, non-prominent Golgi apparatus, abundant glycogen granules, and several cytoplasmic vacuoles with an amorphous or irregular reticular substance [83]. After 14 ds [83], 30 ds [22], and 8 mo [23], the cells exhibit cartilage-like [181,182], zone-associated specific shapes (surface to depth: flattened/ellipsoidal to spherical) [23,39] and form aggregates (normally two to ten cells/cluster, rarely 20 or more) [83]. The cytoplasm of the cells contain one or more prominent nuclei [22], numerous mitochondria and polyribosomes, developed granular endoplasmic reticulum and polyribosomes, and Golgi area, the vacuoles of which contain granules and filaments, with an overall increase in the cell volume [83].

#### 4.2.2. Cell Viability

When stored at 4 °C for 30 ds, the viability of the chondrocytes embedded in alginate does not significantly decrease over the culture period (67% before storage and 60% after storage using high viscosity alginate) [183].

When maintained in culture at 37 °C for 30 ds, the viability of chondrocytes in alginate increases during the period of culture (85% by digestion from cartilage and 95% upon alginate dissolution) [162].

When maintained for 3 mo at 37 °C, the viability of chondrocytes in alginate is stable and of at least 80% throughout the period of culture [184].

There is no significant difference in the viability of chondrocytes kept in a medium with or without serum [112] nor between chondrocytes in monolayer and in alginate cultures [167]. Nevertheless, there is a significant difference in the viability of cells from normal versus OA cartilage [185] and using high [183] versus low viscosity alginate [162].

The viability of chondrocytes also depends on nutrient supply and oxygen, and on the presence of wastes that may diffuse throughout the alginate [137], while normal cell metabolism is not negatively affected by the conditions of encapsulation in alginate.

#### 4.2.3. Cell Proliferation

Significant differences in the levels of cell proliferation exist depending on the source (and relative age) of chondrocytes during the alginate culture (Table 1).

Relative to human cells, for instance [167,187], or under stimulation [167,187], significant increases in proliferation rates were reported for porcine chondrocytes [167,187] at any time of the culture period.

Similarly, significant increases were found in bovine [39,186] or rat [188] chondrocytes during a short culture period, after which there were no significant changes in the DNA content of the cells.

Instead, neither significant increases [27] nor minimal increases [162,184] were detected when using normal human chondrocytes at any time of the culture period, but significant increases [179] were noted in dedifferentiated human chondrocytes during a short alginate culture period, followed by decreases in the DNA contents of the cells [94,97]. In normal dedifferentiated chondrocytes, there was a tendency towards increases in the cellularity of monolayer culture and a tendency towards decreases in alginate culture.

#### 4.2.4. ECM Deposition

ECM is produced by the chondrocytes and can be found in an intracellular location, in the alginate itself, and in the culture medium [137].

ECM in alginate consists of a cell-associated matrix (a pericellular and territorial matrix) and a removed matrix (an interterritorial matrix) [23,27,39]. The pericellular (lacunar) matrix surrounds the chondrocyte plasmalemma as a thin rim rich in PGs but without fibrous proteins (collagens) [189]. The territorial (capsular) matrix adjacent to the pericellular matrix has a fine network characterized by fibrous proteins (collagens) extending around the chondrocytes [189]. The interterritorial matrix surrounds the territorial matrix as the outermost domain characterized by fibrous proteins (collagens) running in parallel and interspersed with PGs [189].

A relative volume of cell-associated matrix (5.2%), removed matrix (91.3%), and chondrocytes (3.5%) can be observed after 13 ds of culture [19,39]. After 30 ds, the relative and absolute volumes occupied by the cell-associated matrix (0.8%), removed matrix (97.2%), and chondrocytes (2%) are nearly identical to those present in the native articular cartilage (cell-associated matrix: 1.7%; removed matrix: 96%; chondrocytes: 2.3%) [102].

The chondrocytes produce higher amounts of aggregating PGs (aggrecan, decorin, biglycan, asporin, lumican, and fibromodulin) in alginate gels than in monolayer cultures [18,190]. The proportion of PG synthesis is 11.31% in chondrocytes, 14.83% in alginate, and 73.86% in the culture medium (24 h labeling at the end of a 28-day culture period) [137].

In alginate, the proportion of cell-associated matrix synthesis is one third (4 h labeling after a 35-day culture period) [27] or one quarter (4 h labeling after a 9-day culture period [102] or 16 h labeling after a 12-day culture period) [19], and the proportion of removed matrix synthesis is two thirds or three quarters.

The deposition of PGs in alginate is either a natural cell-associated matrix deposition, a cell-associated matrix re-deposition, or a deposition of naturally removed matrix. In the first case, most newly synthesized GAGs capable of binding to HA form a mature, functional, HA-binding region (e.g., aggregating PGs) for the chondrocytes [27,189]. Due to interactions between HA and CD44-like HA receptors in chondrocytes, a cell-associated matrix is built on the chondrocyte membrane [46,191,192,193]. Moreover, the concentration of GAGs during cell-associated matrix deposition is similar to that in adult human articular cartilage and is 40-fold higher than in a removed matrix [102]. In the second case, with the matrix removed enzymatically, the addition of exogenous HA and PGs can substitute for endogenous macromolecules and re-assemble a cell-associated matrix within 2 h [193].

With the continued obstruction of CD44–HA interactions, the chondrocyte cell-associated matrix is displaced and the re-assembly of an endogenous cell-associated matrix is inhibited [191]. Therefore, increases in PGs and HA are insufficient to generate a large cell-associated matrix; instead, increases in CD44-like HA receptors and overall HA-binding capacity are required [193]. In the third case, approximately half of the newly synthesized PGs spend less than 24 h in the cell-associated matrix before diffusing in the removed matrix of the chondrocytes [189]. The PGs diffused to the removed matrix during the first 24 h after synthesis are found to lack a functional HA-binding region to keep in the cell-associated matrix [102]. With the gradual saturation of CD44-like HA receptors on the cell membrane after 24 h, more and more aggregating PGs are deposited in the removed matrix.

The accumulation of PG deposition in alginate is controlled by the production of PGs per cell and by the cell density in association with the cell proliferation [178]. The deposition of PGs is gradually accumulated without the effect of the DNA amounts, as seen in a 4-wk study using bovine chondrocytes [39]. The deposition of PGs is more rapidly accumulated with the effect of the DNA amounts, as noted in a 3-wk study using porcine chondrocytes [167]. The mRNA expression of PGs decreases after 4 ds (bovine chondrocytes) [15], but is still detectable after 8 mo of culture using human chondrocytes [194].

Regarding the decomposition of the PG deposition in alginate, the metabolism of PGs is much more dynamic in a cell-associated matrix than in a removed matrix [102,189], with the average half-life of GAGs from the cell-associated matrix being from 15 [189] to 29 ds [102] and that of those from the removed matrix being from 95 [189] to 100 ds [102]. The pool of aggregating PGs has a much longer half-life (2 yr) than that of the non-aggregating PGs that are rapidly lost from the alginate [27,189]. The catabolism of aggregating PGs in a cell-associated matrix is the sole significant contributor to the appearance in the medium of partially degraded GAGs that lose the ability to bind to HA [189].

Alginate has a limited capacity to retain GAGs by lack of a functional HA-binding region and/or partial degradation, as most of the GAGs are found in culture medium [137], depending on their weight and shape [171,195]. Even though alginate does not retain a majority of produced GAGs, the loss of ECM molecules may accelerate the neoformation of cartilage around the implanted bead in vivo [137].

The chondrocytes produce cartilage-specific type-II collagen but not type-I or -X collagen, which are produced when the cells lose their phenotypic stability [23,171]. The proportion of collagen deposition in chondrocytes and alginate exceeds 97.5% and is below 2.5% in culture medium after a 28-day period [39]. The deposition of fibrous proteins in alginate consists of a normally differentiated deposition of type-II/-IX/-XI collagen and of an abnormally differentiated deposition of type-I/-X collagen. In the first case, type-II/-IX/-XI collagen expression is detected at all times of the culture period (7/15/28 ds) both in the cell-associated matrix and in the removed matrix in relative proportions (95/1/3) similar to those in the articular cartilage in its cell-associated matrix [39]. A high proportion of cell-associated matrix deposition/removed matrix deposition can be found for type-II/-IX/-XI collagen and the proportion in type-II or -IX collagen is higher than that in type-XI collagen [39]. In the second case, the accumulation of type-X collagen is very weak and limited to the surface of the alginate [35], indicating that hypertrophic differentiation and endochondral ossification (transdifferentiation) occurs in this region [35,39]. Type-I collagen is synthesized in small amounts by flattened fibroblast-like cells (dedifferentiation) at the edge of alginate, and does not become incorporated as heterotrimers or homotrimers in the matrix [39]. The accumulation of fibrous protein deposition in alginate is also controlled by the production of collagen in each cell and by the cell density, which relates to cell proliferation. The deposition of collagens increases during the culture period with or without the effect of cell proliferation, both in the cell-associated matrix and removed matrix [39].

The mRNA expression of type-II collagen decreases from the first day, while that of type-I collagen increases over an 8-day culture period [15]. The mRNA expression of type-II collagen increases, instead, over a 3-wk culture period, while that of type-I collagen decreases [167]. This is not conflicting, as the former observation has been reported when using normal chondrocytes in alginate while the later was noted in dedifferentiated chondrocytes [34,84]. In addition, the mRNA expression of type-I/-II collagen at 21 ds is similar to that on day 1 of culture [196].

Hydroxy-lysyl pyridinoline (pyridinoline) is the major crosslinking residue (>93%) in mature type-II collagen [35,39,197]. Lysyl-pyridinoline (deoxy-pyridinoline) is slightly present and forms the crosslinks of type-IX collagen [39,197,198]. Pyridinium crosslinks are detected both in the cell-associated matrix (90%) and in the removed matrix (10%) at all times, while the content of crosslinks increases with time [35,39]. The ratio of pyridinoline to deoxy-pyridinoline shows a moderate drop with time in the removed matrix, but an increase with time in the cell-associated matrix [39]. Of note, the crosslinking can be blocked (100%) by β-aminopropionitrile (BAPN) without affecting cell proliferation and PG deposition [35,199].

The collagen network in the mature cartilage is detected with an extremely slowly turnover in vivo (t_1/2_ > 100 yr) [200], while the minor collagens in the remodeling or reorganization of the fibrillar network possibly have a more rapid turnover in vitro [39].

Alginate has a powerful capacity to retain collagen and the ECM in alginate is rich in collagen fibers [137], depending on the weight and shape of the molecules (collagens) [171,195]. The concentration of collagen in culture medium does not reach a measurable level, but a small proportion of the newly synthesized collagens possibly escape from alginate over the culture period [39].

The deposition of cell-associated matrix and of removed matrix in vitro can be separated using mild centrifugation conditions (100× *g* for 10 min at 4 °C [39,102]/900 rpm for 9 min [27]/100 g) [35] as the cell-associated matrix is firmly anchored to the chondrocytes via HA strands, while the removed matrix is not. This approach is employed to detect the relationships between the PGs and collagens of the cell-associated matrix and the removed matrix in alginate in vitro.

#### 4.2.5. Cell Redifferentiation

The use of alginate is useful not only to maintain a differentiated phenotype in the chondrocytes, but also to restore a normal phenotype [25] in dedifferentiated chondrocytes, even from OA cartilage [201].

The culture of dedifferentiated chondrocytes in alginate leads to an increased expression of PGs and type-II collagen and to a decreased expression of type-I/type-X collagen [34,94,97,196,202,203].

**Figure 4:**
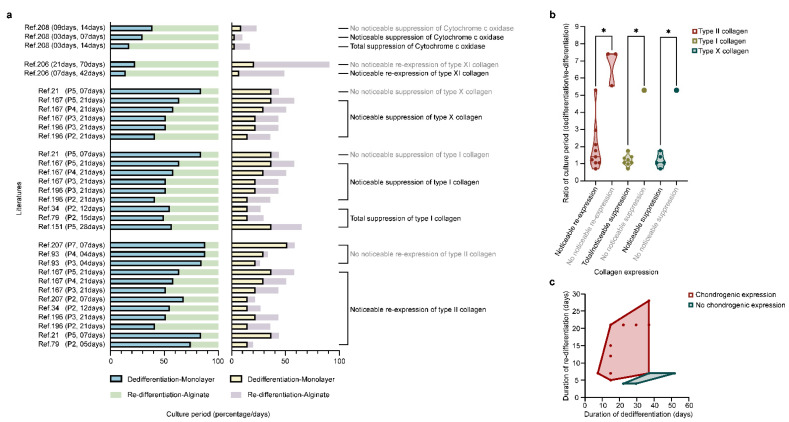
Relationship between the cell redifferentiation abilities. (**a**) Modification of cell expression associated with the relative/absolute relationship between dedifferentiation and redifferentiation (duration of passage: 7.4 ds, according to [21,34,79,93,151,167,196,206,207,208]). (**b**) Ratio (dedifferentiation/redifferentiation) for collagen expression (* indicates statistically significant differences between groups). (**c**) Relationship between chondrogenic expression (PGs, type-II/-I/-X/-III collagen) and culture period (dots represent independent publications matching the parameters of duration of chondrocyte redifferentiation versus differentiation) (created with Prism (**a**,**b**) and Past (**c**)).

Redifferentiation permits the occurrence of zonal differences, with the re-deposition of SZP and clusterin (superficial zone) and of COMP and GAGs (deep zone) that disappeared during dedifferentiation [38]. Redifferentiation also re-induces the expression of CD44-like HA receptors that diminished during dedifferentiation [99] and restores the mRNA expression of TβRII and the subsequent TGF-β response, suppressed during dedifferentiation [100]. Importantly, increasing the duration of the monolayer culture leads to a decreased redifferentiation potential [84,96,204,205], while increasing that of alginate culture leads to an increased redifferentiation ability (Figure 4) [206,207,208].

**Figure 5:**
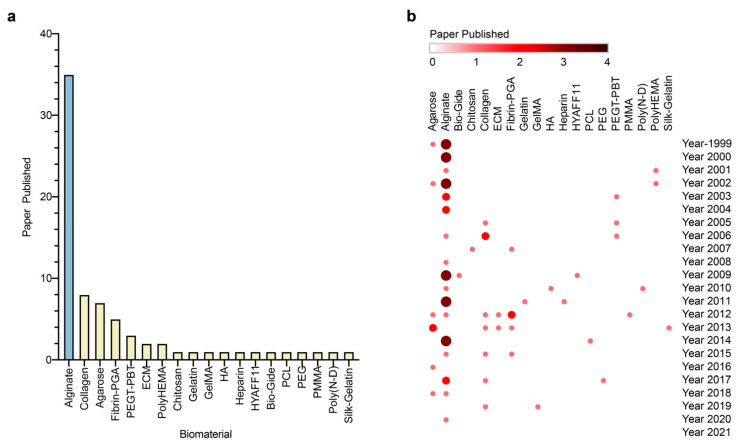
Use of alginate for chondrocyte redifferentiation versus other materials. Biomaterials used for chondrocyte redifferentiation in total (**a**) and annual (**b**) publications. PGA, poly(glycolic acid); GelMA, gelatin methacryloyl; HA, hyaluronic acid; PCL, poly(ε-caprolactone); PEG, polyethylene glycol; PEGT-PBT, polyethylene glycol terephthalate/polybutylene terephthalate; PMMA, poly(methyl methacrylate); Poly(N-D), poly(NaAMPS-co-DMAAm); PolyHEMA, poly-(2-hydroxyethyl methacrylate); created with Prism (**a**) and Morpheus (**b**).

Decreasing the chondrocyte seeding density in the monolayer culture (3 × 10^4^ to 3.5 × 10^3^ cells/cm^2^, duration of passage: 4 ds versus 21 in alginate) [209] or in alginate (10^6^ to 10^4^ cells/mL, duration of passage: 28 ds versus 3 in the monolayer culture) [33] improves their redifferentiation capacity. This needs to be taken into account, as dedifferentiated cells have been wrongly seen as differentiated cells [166,167,188,210,211]. Chondrocyte redifferentiation in alginate [212] versus other materials (Figure 5) involves several pathways identified using physicochemical and biological stimuli (hypoxia, hydrostatic pressure, oxygen, seeding density, co-culture, temperature, and growth factors/genes, such as bone morphogenetic proteins (BMPs), TGF-β, insulin-like growth factor I (IGF-I), basic fibroblast growth factor (FGF-2), platelet-derived growth factor (PDGF), EGF, platelet-rich plasma (PRP), platelet lysate, ECM), or inhibitors of calcineurin, Rho activation, activin A, transglutaminase 2, and extracellular signal-regulated protein kinase (ERK1/2) [33,36,201,213,214,215,216,217,218,219,220,221,222,223,224,225,226,227,228,229,230,231,232,233,234,235,236,237,238,239,240,241,242,243,244,245,246,247,248,249,250,251].

Chondrocyte redifferentiation is generally accompanied with an enhanced expression of the cartilage-specific sex-determining region Y-type high mobility box 9 (SOX9) transcription factor [252,253] via the p38 MAPK (a mitogen-activated protein kinase related to the c-Jun N-terminal kinase (JNK)) signaling pathway [254], activating ECM synthesis (PGs, type-II collagen) [255]. Redifferentiation may occur via the TGF-β/TβRII, FGF-2/FGF receptor 3 (FGFR3), and protein kinase C (PKC), ERK, and focal adhesion kinase (FAK) signaling pathways [100,202,256,257].

### 4.3. In Vivo Studies

Engrafted alginate–chondrocyte systems have been tested in vivo and the subsequent findings of cartilage structure formation were demonstrated using either the extra- or intra-articular application of the systems (Table 2) [82,138,166,211,258,259,260,261,262].

#### 4.3.1. Extra-Articular Implantation

Chondrocytes engrafted in alginate and extra-articularly implanted in vivo can be detected at the site of implantation for the entire period of observation with increased cell densities over time in lapine [260], bovine [138], and porcine models [166].

Chondrocytes become surrounded with basophilic ground substance between 4 and 20 wk in vivo [138,258,260] with an accumulation of ECM deposition for at least 6 wk [166]. PGs and type-II collagen [259] can be detected in the grafts (4–20 wk/4–28 wk) [138,258,260] with increases in deposition also for up to 6 wk [166]. The mRNA expression of PGs and type-II collagen decreases over time (wk 2/4/6) with a short period of up-regulation (wk 2/4) followed by a period of down-regulation (wk 6) [166]. The mRNA expression of type-X and -I collagen remains stable to nearly undetectable levels over time (wk 2/4/6) [166].

The grafts show an irregular, gradually pearly opalescence, firm but flexible to palpation, and resembling the native articular cartilage at the time of harvest [138,166,258,259,260]. They are surrounded by a thin layer of fibrous tissue characterized by a vascular network relating to nutritional supply [138,258,259,260]. They exhibit an increasing weight after incubation for 12 wk [258] that remains stable until after 38 and 20 [259] wk.

#### 4.3.2. Intra-Articular Implantation

Chondrocytes intra-articularly transplanted in vivo can be detected in the repair tissue during the whole period of observation without evidence of strictly increased or reduced cell densities in lapine [82] and ovine [211] models. Of note, chondrocytes labelled via transfection methods may be varied during cell density detection, as the number of transfected cells may differ from that of non-transfected cells in alginate [82,211].

There is no indication of the migration of engrafted chondrocytes in the host articular cartilage or of the integration of the regenerated cartilage with the host articular cartilage [82]. The is also no evidence of the accumulation of basophilic ground substance deposition nor of increases in PG deposition without the formation of fibrous tissue, even though these were detected respectively [82,261].

Over a follow-up period of about 6 yr, there is a remarkable difference between favorable clinical determination (Western Ontario and McMaster Universities Osteoarthritis Index (WOMAC) score; visual analog scale (VAS)) and the moderate to poor MRI findings (Magnetic Resonance Observation of Cartilage Repair Tissue (MOCART) score) [262]. Magnetic resonance imaging (MRI) scores do not show satisfactory results of cartilage lesion regeneration, while clinical scores cannot predict the long-term durability of outcomes, even though severe clinical deterioration or adverse reactions are not observed [262]. Additionally, the application of alginate with agarose for ACI offered safe and durable cartilage repair over a period of 18 yr [263].

#### 4.3.3. Implantation Methods

Implantation is performed via injection or arthrotomy to deliver large amounts of isolated chondrocytes as a means to promote engraftment and cartilage formation [258]. Higher seeding densities are commonly employed in vivo with stable concentrations of alginate and gelling solution compared with the conditions used in vitro (Table 3).

At implantation, a rapid gelation may limit the minimal invasion and relatively adequate mixing, which may be retarded by temperature dropping (4 °C) [120,258] and with cations (CaSO_4_/Na_2_HPO_4_) (Table 4) [264].

Clinical implantation procedures include the management of chondrocytes for intra-articular application (harvesting, expansion, encapsulation), the preparation of the defect (cleaning, regularization), the implantation of the engrafted system, and the possible use of a periosteal flap sealing [262].

In the case of an acellular implantation, in situ gelation is preferred at the time of the operation without additional fixation of the augmentation site [163].

### 4.4. Overview and Limitations

While chondrocytes are known to better maintain their phenotype in alginate versus monolayer cultures, their proliferation for natural turnover and ECM deposition in this 3D conformation are still insufficient for effective cartilage regeneration. Therefore, the addition of appropriate exogenous repair stimuli may improve the capability of chondrocyte proliferation and ECM deposition in alginate culture [80,144,265].

## 5. Alginate, Chondrocytes, and Biological/Physicochemical Stimulation for Articular Cartilage Regeneration

Experimental conditions have thus been further expanded to enhance the potential of chondrocytes in alginate via a number of stimuli to further enhance the processes of cartilage regeneration.

Such stimuli include biologics (growth factors), physicochemical components (tension, shearing force, perfusion, hydrostatic pressure, oxygen tension, osmotic pressure, ultrasounds), and other types of stimulation (rhein, ascorbic acid, chlorogenic acid, cyclodextrin polysulfates, flavonoid compound icariin, BAPN).

Most investigation has been performed in experimental studies in vitro and partly in vivo, as detailed below.

### 5.1. In Vitro Studies

#### 5.1.1. Biological Stimulation

Biological stimuli include various factors, most particularly growth factors, such as IGF-I, TGF-β, FGF-2, osteogenic protein 1 (OP-1), and BMP-2 (Table 5) [19,80,84,185,193,266,267,268,269,270,271,272,273,274].

IGF-I is a key intrinsic factor involved in the articular cartilage homeostasis [275] associated with degeneration and regeneration [145]. Chondrocyte viability and proliferation is minimally influenced by the addition of IGF-I in the absence of serum, while PG and type-II collagen deposition is slightly affected, regardless of the cell numbers [185,268]. Chondrocyte viability and proliferation is also minimally affected by the addition of IGF-I in the presence of serum, but PG and type-II collagen deposition is significantly increased, regardless of the cell numbers [19,269,270]. For individual cells, PG synthesis increases for a short time and then decreases in association with the activation of anabolic (PI3K activity) and catabolic pathways upon the addition of IGF-I in the absence of serum [276].

TGF-β is also an intrinsic factor that is secreted as a latent high molecular weight complex in the articular cartilage, becoming activated upon complex dissociation [84]. Chondrocyte proliferation in alginate is not affected by the addition of TGF-β2 in the presence of serum [84,270], but PG synthesis is significantly increased, regardless of the cell numbers [19]. PG deposition is associated with ECM assembly, with the reduction of relative volume in cell-associated matrix as a result of external TGF-β2 stimulation with serum [19], which can be blocked gradually during ECM formation [84].

FGF-2, a member of the multifunctional fibroblast growth factor family, displays mitogenic activities in chondrocytes [268,277,278]. Chondrocyte viability is not altered by the addition of FGF-2, yet proliferation is stimulated regardless of the presence of serum, while PG deposition decreases in a dose-dependent manner in individual cells [268,270]. The synthesis of PGs is slightly affected by FGF-2, showing an increased degradation regardless of the cell numbers [268]. FGF-2 can also block the improved PG synthesis mediated by IGF-I and OP-1 [268].

OP-1 (BMP-7) is an anabolic growth factor of the TGF superfamily expressed in the articular cartilage [185,271,274]. The addition of OP-1 results in both increased cell proliferation and PG deposition in the presence or absence of serum, regardless of the cell numbers [93,185,193,267,268,271,272,273]. For individual cells, PG synthesis and type-II collagen deposition are rarely affected by the addition of OP-1 and differences between the deposition and synthesis of PGs have been related to a diminished catabolism [273].

BMP-2 plays key roles in chondrocyte differentiation and ECM maturation [80]. The synthesis of PGs and type-II collagen by individually dedifferentiated chondrocytes is significantly increased by the addition of BMP-2 in the presence of serum [80]. Interestingly, such stimulating effects may be affected by different phenotypes [84,271]. Therefore, it appears to be important to take these observations into careful account, as dedifferentiated cells do not represent cells with a normal phenotype [80,98,271].

#### 5.1.2. Physicochemical Stimulation

The process by which mechanical loading influences the metabolism of the chondrocytes in alginate is called mechanotransduction [130]. The various types of mechanical stress applied to alginate where the chondrocytes are embedded include tension, shearing force, and hydrostatic pressure (Table 6) [20,23,36,219,279,280,281,282,283,284,285,286,287,288,289,290,291,292,293,294], and these can influence each other [280,281].

Regarding tension, static compression may affect the deformation of chondrocytes depending on the concentration of alginate, with a durability of cell distortion parallel to the viscoelasticity of alginate [130]. Static compression is known to decrease the synthesis of PGs in the chondrocytes, while cyclic compression increases it, and sulfate incorporation is affected by mechanical compression [20]. The effect of compression is linked to the duration, intensity, and frequency of the tension, to the incubation period before and after mechanical stimulation, and to the chondrocyte phenotype [20,282]. The increasing effect of shearing force is associated with the duration and velocity of the perfusion method, with the incubation period before and after stimulation, and with the chondrocyte phenotype [281,283,284]. The distribution of PGs is more uniform in perfusion culture, and a higher PG deposition can be found both in the outer regions of the alginate and in the culture medium [281,283]. The positive effect of hydrostatic pressure is also related to the duration, intensity, and frequency of stimulation, to the incubation time before and after stimulation, and to the chondrocyte phenotype [279,285].

The articular cartilage is a physiologically hypoxic tissue [295], with a gradient of oxygen tension ranging from about 10% [286] (7.5%) [203] at the cartilage surface to less than 1% [203,286] in the deepest layers. In conditions where the oxygen tension is less than 0.1% [286] or more than 50% [203], the metabolism of the chondrocytes in alginate is severely compromised. Dedifferentiated chondrocytes are sensitive to hypoxia [36,203,219,288], in contrast to differentiated [286,287] and septal chondrocytes [289]. Cell viability and proliferation are independent from hypoxia [36,288], which may however increase the synthesis and deposition of PGs and type-II collagen in dedifferentiated chondrocytes, regardless of the cell numbers (Table 6) [36,203,219,288].

Extracellular osmotic pressure is mainly determined by the local PG concentration in the different articular cartilage zones, ranging from about 350 mOsm in the superficial zone to about 450 mOsm in the middle zone [296,297]. The effect of hypotonic (<300 mOsm) or hypertonic (>500 mOsm) medium changes for chondrocytes embedded in alginate is associated with the duration and magnitude of stimulation and with the culture medium [290,291,292]. Furthermore, chondrocytes are more sensitive to hypotonic than to hypertonic pressure, with mostly negative effects (Table 6).

The effects of ultrasounds on chondrocytes in alginate are related to the duration, intensity, and frequency of stimulation and to the cell phenotype, but without strong effects on cell viability and proliferation or on ECM deposition (Table 6) [293,294].

#### 5.1.3. Other Stimuli

Chondrocyte proliferation and ECM deposition in alginate may also be influenced by rhein enhancing the PG deposition [298], ascorbic acid increasing the levels of cell proliferation, but without effect on type-II collagen deposition [299], chlorogenic acid promoting higher levels of cell proliferation and of total PG deposition [300], cyclodextrin polysulfates enhancing the total synthesis/deposition of PGs and of type-II collagen [301], flavonoid compound icariin increasing cell proliferation and the synthesis/deposition of PGs and of type-II collagen [302], or even BAPN stimulating the total deposition of PGs and of type-II collagen [199] while lidocaine may inhibit such effects in the cells [303,304,305].

### 5.2. In Vivo Studies

Thus far, there is relatively little information on applying such stimuli to chondrocytes in alginate in vivo, since adapted interventions are not easy to control, and the measurements of target chondrocytes are difficult to perform. For instance, the application of growth factors to chondrocytes in alginate as recombinant peptides (PDGF) [306] or in the form of genetic sequences (FGF-2, IGF-I), delivered via nonviral gene vectors as single sequences [145,307] or in combination [308], promoted effective cartilage repair in rabbit osteochondral defects for at least 14 wk, with enhanced levels of cell proliferation and of PG and type-II collagen deposition in vivo. Additionally, hypoxia [309] and hyperbaric oxygen [306] were reported to significantly increase total PG and type-II collagen deposition in encapsulated chondrocytes in vivo.

### 5.3. Overview and Limitations

Overall, stimulating factors have no negative effects on cell viability, yet in certain conditions (OA/dedifferentiation), cell viability under specific stimulation may decline with time. For differentiated chondrocytes, few stimulation factors (OP-1) have positive effects on both cell proliferation and ECM deposition (PGs, type-II collagen), regardless of the cell numbers, but only short-term findings are available thus far. Under suitable stimulation, ECM deposition and/or cell proliferation may be promoted in alginate gel, although the duration of these effects may not be maintained over time to achieve adapted, permanent cartilage regeneration.

## 6. Alginate and Progenitor Cells for Articular Cartilage Regeneration

Progenitor cells, such as mesenchymal stromal cells (MSCs), have further been manipulated as a naturally chondro-reparative source of cells capable of developing a chondrocyte phenotype (morphology, cell viability, ECM deposition) [8] via entrapment in alginate as a means to improve the processes of cartilage regeneration [310,311]. Recently, dental pulp stem cells (DPSCs) also gained interest as an alternative source of chondrogenically competent progenitor cells for cartilage repair [312]. Induced pluripotent stem cells (iPSCs) represent another attractive source of progenitor cells for cartilage tissue engineering [313,314] that may also be generated from human OA chondrocytes [315]. Interestingly, the transfection of iPSCs using a lentivirus–TGF-β1 construct [315], co-cultured with chondrocytes [315,316], promoted the chondrogenic differentiation of iPSCs using an alginate matrix.

### 6.1. In Vitro Studies: Differentiation after Encapsulation

The chondrogenic differentiation of progenitor cells is induced by interactions between alginate and the cells in vitro [8,89], and may be further induced by the culture (chondrogenic) medium as well as by a number of growth factors (IGF-I, TGF-β1/-β2/-β3, BMP-2/-7) [317,318,319,320,321,322], low-intensity ultrasounds [323], platelet-rich concentrate [324,325,326], and by a combination of such factors. The viability of progenitor cells in alginate increases early on in culture and then decreases over time [324], possibly due to a switch towards phenotype differentiation in the whole differentiating cell population. The mRNA expression of PGs and type-II collagen is detectable over the whole period of culture (ds 2 [8]/6 [321]/14 [323]/21 [322,325]/28) [313] with increases at the end of the period of culture (ds 14 [320]/21 [327]/24) [319,324], regardless of the cell numbers. Type-I collagen synthesis may increase [320] or not [322] in the presence or absence of BMP-2, respectively, regardless of the cell numbers [318,320,322]. Type-X collagen synthesis [322,324] and deposition [318] occurs with an increase over time in culture, regardless of the cell numbers. Overall, while progenitor cells in alginate commit towards the chondrocyte phenotype, they ultimately enter hypertrophic differentiation and endochondral ossification as the final form of differentiation after encapsulation (Table 7) [8,313,314,316,318,319,320,321,322,323,324,325,326,327,328,329,330].

### 6.2. In Vivo Studies: Encapsulation after Differentiation

In vivo, cells undergo the mRNA expression of PGs and type-II collagen with an increase over time depending on the number of cells provided [328]. Interestingly, while type-I and type-X collagen synthesis occurs at the level of individual cells [328], the deposition of these markers for total cells remains stable at a low level, without sign of dedifferentiation or transdifferentiation (Table 7).

### 6.3. Overview and Limitations

Overall, the outcomes achieved using progenitor cells in alginate in terms of effective cartilage regeneration parallel those supported when using chondrocytes [331,332,333,334,335,336,337,338]. Again, however, the adapted cartilage regeneration processes are not sufficiently addressed with either of these sources of cells to support the long-term healing of this tissue. Genetic enhancement appears to be a potent, emerging approach to amplify the outcomes for lasting regeneration [145] and to promote the spatial and temporal control of cartilage regeneration.

## 7. Alginate Combined with Gene Therapy for Articular Cartilage Regeneration

Gene therapy is an attractive approach that uses genes to prevent or cure diseases, and gene therapy for articular cartilage regeneration is based on the overexpression of chondro-reparative agents, such as growth or transcription factors, or of molecules that may impede joint destruction via gene delivery vectors [5].

Nonviral vectors (plasmids, cationic liposomes, peptides, DNA-ligand complexes, gene-gun systems) [5,32,339] as well as viral vectors (adenoviral vectors, herpes simplex virus (HSV) vectors, retroviral vectors, lentiviral vectors, recombinant adeno-associated viral (rAAV) vectors) [108,110,340] have been employed as gene transporters for the exogenous expression of therapeutics to improve cartilage regeneration.

**Figure 6:**
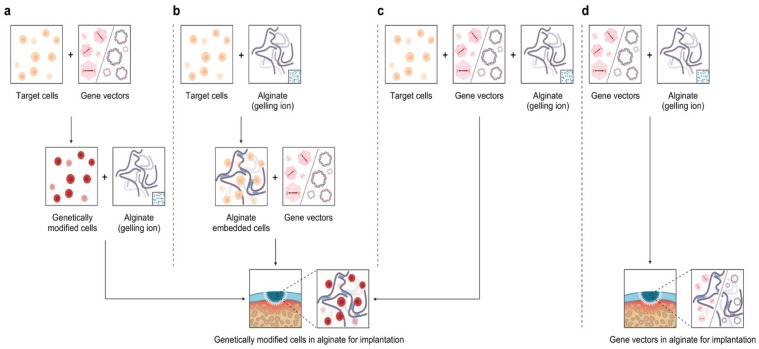
Strategies using gene therapy combined with alginate for articular cartilage regeneration. The approaches include (**a**–**c**) the indirect, cell-associated encapsulation of genetically modified cells in alginate and (**d**) the direct, cell-free formulation of gene transfer vectors in alginate, both as potential implantable platforms in articular cartilage defects. (**a**) Genetic modification before alginate encapsulation [68]. (**b**) Genetic modification after alginate encapsulation [343]. (**c**) Genetic modification with alginate encapsulation (gene-activated matrix) [344,345]. (**d**) Gene vectors with alginate encapsulation [342]. Created with BioRender.com.

The strategies developed to combine gene therapy with the use of alginate thus far include the encapsulation of genetically modified cells in alginate (indirect, cell-associated approach) [5,68,145] and the formulation of gene transfer vectors in alginate (direct, cell-free approach) (Figure 6) [5,341,342,343,344,345].

### 7.1. Indirect Encapsulation of Genetically Modified Cells in Alginate

Articular chondrocytes that are genetically modified via nonviral vectors to overexpress IGF-I or FGF-2 [68,145,341,346] and then embedded in alginate undergo significant, continuous increases in cell proliferation, especially when applying IGF-I (Table 8) [145,341,346]. Upon IGF-I overexpression, an increase in total PG deposition is observed after 3/14 wk in vivo without the modification of the total deposition of type-II collagen [145], while type-II collagen and PG deposition increases at variable levels upon FGF-2 overexpression [68], in agreement with the effects of these growth factors [268,270]. Related results are noted when applying alginate with genetically modified progenitor cells from the bone marrow or from the adipose tissue upon the adenoviral or lentiviral gene transfer of IGF-I [346] or TGF-β [146,347,348], leading to the deposition of PGs and type-II collagen (Table 8).

### 7.2. Direct Formulation of Gene Transfer Vectors in Alginate

The delivery of gene transfer vectors via alginate (scaffold-guided gene transfer) has been first attempted using reporter gene constructs, such as coding for the Escherichia coli β-galactosidase (*lacZ*) marker gene (Table 8) [341]. In this work, the viability of MSCs in vitro was not impaired when using an rAAV-*lacZ* formulated in alginate, as expected with a reporter gene. The treatment also did not alter the deposition of PGs and type-II collagen in MSCs, showing the safety of the approach [341].

Recently, the application of alginate formulating an rAAV vector carrying the therapeutic IGF-I gene within sites of chondral defects led to significant increases in the levels of local cell proliferation in the lesions as well as in the deposition of PGs and type-II collagen over an extended period of one yr in vivo without deleterious (toxic, immunogenic) responses to the treatment (Table 8) [342].

It remains to be seen, however, whether such a therapeutic alginate–rAAV composite system can further improve cartilage regeneration over much longer periods of time and whether the mechanical functions in the repair tissue of the treated lesions will be restored to those of the natural, hyaline cartilage.

Such an approach nevertheless shows the high value of using alginate in combination with gene therapy to improve the processes of cartilage regeneration especially in settings in vivo.

## 8. Emerging Alginate Systems with Improved Mechanical Properties for Articular Cartilage Regeneration

While alginate has been previously regarded as a compound lacking sufficient mechanical strength to support adapted cartilage regeneration, work has been performed and advances were accomplished to overcome this limitation for in vivo and clinical applications [116,136,164,326,346,349,350,351,352,353,354,355,356,357,358,359,360,361,362,363,364,365,366,367,368,369,370,371,372,373,374,375,376,377,378,379].

With this goal, alginate was either chemically modified or engineered (sulfhydrylation, alkylation, dopamine modification) [349,354,359,361,376] or combined with other materials or compounds in order to generate mechanically stronger composite systems adapted for cartilage engineering [116,326,346,350,351,352,353,355,356,357,358,360,362,363,364,365,369,370,371,372,379].

Materials and compounds used to form composites with alginate include collagen [116,352], PRP [326], bovine cartilage matrix [346], agarose [352], polyvinyl alcohol (PVA) [364], poly(ε-caprolactone) (PCL) [365], N,O-carboxymethyl chitosan/polyphosphate (N,O-CMC-polyP) [363], polyacrylamide (PAAm) [355,362,365], poly(L-lactic acid) (PLLA) [371], hyaluronic acid (HA) [360], polymethacrylate (PMA) [350], nanocellulose [351,358,379], poly(2-ethyl-2-oxazoline) (PEOXA) [351], chondroitin sulfate [357], polyethylene glycol (PEG) with fibrin [370], and chitosan [353,356].

Overall, the mechanical properties of the expanded alginate hydrogels reported in these composites were adapted to those of the articular cartilage and often stronger than when using alginate alone [136,164,366,367,368,373,375,377,378], especially in cell-associated experimental conditions in vitro and in vivo for improved cartilage formation [116,366,376,379].

Such upgraded systems, however, will need to be further assessed for their benefits in translational models in vivo, while they may be also investigated in approaches combined with gene therapy.

## 9. Conclusions and Perspectives

In light of the abundant literature on the competent application of alginate, this compound represents a promising biomaterial in approaches that aim to enhance articular cartilage regeneration [342] besides other (natural or synthetic) hydrogel materials (chitosan, hyaluronic acid, chondroitin sulfate, collagen, fibrin, gelatin, poly(lactide-co-glycolide), polyethylene glycol, poly(N-isopropylacrylamide-coacrylic acid, etc.) extensively described elsewhere [119] and also used for this purpose, although to a prospectively more limited extent [119].

The use of alginate combined with a controlled chondro-reparative gene delivery system may further improve the healing responses of injured articular cartilage at the site of damage as off-the-shelf systems for therapy. However, a number of challenges remain to be addressed when using this material for such a purpose.

First, the presence of certain charged polyanions in alginate [27] may promote the formation of an abnormal collagen ultrastructure, termed segment-long-spacing (SLS)-like crystallites, in the ECM of chondrogenic cultures [380,381,382,383,384].

**Figure 7:**
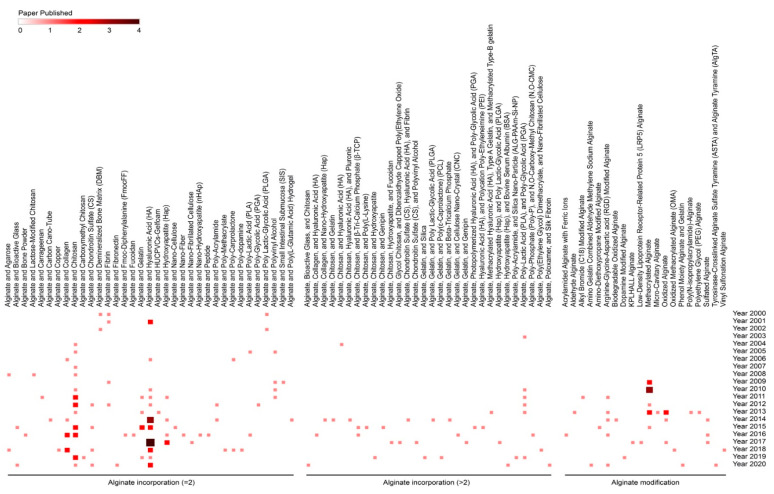
Enhanced cell adhesion with alginate incorporation and alginate modification (yearly distribution of the number of publications); created with Morpheus.

Second, cells may insufficiently adhere to the surrounding alginate, leading to impaired cell viability, proliferation, ECM deposition, and differentiation [113,385]. The incorporation of additional compounds (calcium phosphate [127], lactose-modified chitosan) [356] and the modification of alginate (arginine-glycine-aspartic acid, i.e., RGD) [386] may address these issues, leading to hybrid hydrogels with improved mechanical properties and supporting a better cell phenotype [162,387], and creating a more suitable surface for RGD-mediated cell attachment via the integrins (α5β1 and α5β3 integrins) [388] found on chondrocytes and progenitor cells (Figure 7) [389,390].

Finally, the use of alginate may lead to the generation of a fibrous tissue (fibrocartilage) composed of type-I collagen instead of a natural hyaline cartilage with type-II collagen [97,391]. This might be partly addressed by using ultra-purified alginate (UPAL alginate) and ultra-purified low endotoxin alginate (UPLE alginate) [295].

Other approaches to tackle these issues include the use of co-culture systems in alginate (chondrocytes/progenitor cells) [392,393,394] and of 3D printing techniques with multi-material incorporation [379,395,396] or alginate modification [397,398], as performed in the presence [379,395] or absence [352,399] of cells with nanofibrillated cellulose (NFC) [379,396,400] and PCL [395,401,402].

**Figure 8:**
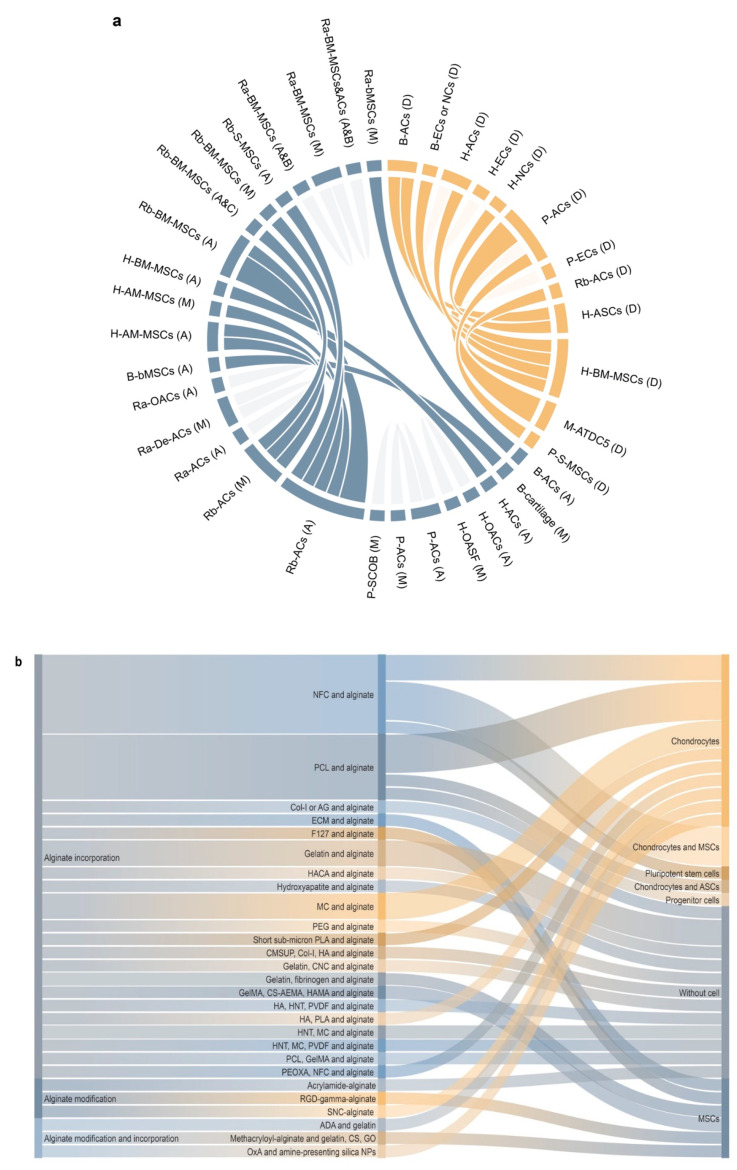
Co-culture systems and 3D printing techniques. (**a**) Co-culture systems using alginate. The orange part presents cells mixed, encapsulated in alginate, and then maintained in culture. The blue part presents cells encapsulated in alginate, mixed, and then maintained in culture. The deep cords show combinations of chondrocytes and progenitor cells (A) in alginate, (M) in monolayer, (C) in collagen, (B) in bioglass, and (D) in direct contact (ACs, articular chondrocytes; AM-, amniotic membrane; B-, bovine; BM-, bone marrow; bMSCs, bone marrow mesenchymal stromal cells; Cartilage, zonal cartilage tissue; De-ACs, dedifferentiated articular chondrocytes; H-, human; MSCs, mesenchymal stromal cells; OACs, osteoarthritic articular chondrocytes; OASF, osteoarthritic synovial fibroblasts; P-, porcine; Ra-, rat; Rb-, rabbit; S-, synovium; SCOB, subchondral osteoblasts). (**b**) Use of alginate as a bioink in 3D printing (ADA, alginate-di-aldehyde; AG, agarose; Alginate SNC, alginate sulfate nano-cellulose; ASCs, adipose-derived stromal cells (progenitors); CMSUP, calcium-magnesium silicate ultrafine particles; CNC, cellulose nanocrystals; Col-I, type-I collagen; CS, chondroitin sulfate; CS-AEMA, chondroitin sulfate amino ethyl methacrylate; ECM, extracellular matrix; F127, Pluronic F127; GelMA, gelatin methacrylamide; GO, graphene oxide; HA, hyaluronate; HACA, catechol modified hyaluronic acid; HAMA, hyaluronic acid methacrylate; HNT, halloysite nanotube; MC, methyl cellulose; MSCs, mesenchymal stromal cells; NFC, nanofibrillated cellulose; NPs, nanoparticles; OxA, oxidized alginate; PCL, polycaprolactone; PEG, poly(ethylene glycol); PEOXA, poly(2-ethyl-2-oxazoline); PLA, polylactic acid; PVDF, polyvinylidene fluoride; RGD-gamma-alginate, gamma-irradiated alginate with RGD peptides); created with Flourish.

All these novel concepts may as well be combined with gene vector delivery using optimally identified chondro-reparative genes as single entities or as simultaneous genetic treatments to achieve optimal effects in cartilage regeneration (Figure 8).

Overall, the exploitation of alginate in the field of cartilage tissue engineering is progressing towards mimicking the natural in vivo microenvironment for improved treatments in patients in a close future.

## Figures and Tables

**Table 1:** Differences of chondrocyte proliferation in vitro.

Origin	Phenotype	Species	Age	Proliferation	Evaluation	Refs.
non-human articular cartilage	normal	bovine	<5 wk	2.8-fold increase	0–20 ds	[186]
		bovine	<5 wk	constant	20–40 ds	[186]
		bovine	14–18 mo	6.9-fold increase	0–21 ds	[39]
		bovine	14–18 mo	constant	21–28 ds	[39]
		porcine	24 wk	60-fold increase	7–28 ds	[187]
	dedifferentiated	rat	4–6 wk	2.5-fold increase	0–40 ds	[188]
		rat	4–6 wk	constant	40–60 ds	[188]
		porcine	10–12 mo	2.7-fold increase	7–21 ds	[167]
human articular/nasal septal cartilage	normal	TKA patient	N/A	no increase	0–20 ds	[162]
		healthy donor	N/A	no increase	0–107 ds	[184]
		healthy donor	14 yr	minimal increase	0–10 ds	[27]
		healthy donor	14 yr	no increase	10–19 ds	[27]
		healthy donor	66 yr	minimal increase	0–8 ds	[27]
		healthy donor	66 yr	no increase	8–19 ds	[27]
	dedifferentiated	TKA patient	N/A	4-fold decrease	1–35 ds	[97]
		healthy donor	5–63 yr	minimal decrease	2–7 ds	[94]
		septoplasty patient	N/A	2.5-fold increase	0–14 ds	[179]
		septoplasty patient	35 yr	1.6-fold increase	0–7 ds	[118]
		septoplasty patient	35 yr	2-fold increase	0–14 ds	[118]

Abbreviations: TKA, total knee arthroplasty; N/A, not announced.

**Table 2:** Implantation of alginate–chondrocyte systems.

Approach	Model	Evaluation	Cartilage Regeneration	Fibrous Tissue	Refs.
extra-articular	dorsal s.c. tissue (nude mouse)	6–12 wk	+	N/A	[258]
	dorsal s.c. pocket (athymic mouse)	8–12 wk	+	N/A	[138]
	dorsal s.c. tissue (nude mouse)	14–38 wk	+	minor	[259]
	dorsal s.c. tissue (SCID mouse)	2–6 wk	+	-	[166]
	gluteus muscle (rabbit)	4–20 wk	+	N/A	[260]
intra-articular	femoral condyle OCDs (rabbit)	4–24 wk	+	N/A	[261]
	trochlear groove OCDs (rabbit)	1–4 wk	minor	major	[82]
	femoral condyle, patella, and trochlea CDs (human)	1–8 yr	N/A	N/A	[262]
	femoral condyle and trochlea OCDs (sheep)	1–21 ds	-	+	[211]

Abbreviations: s.c., subcutaneous; SCID, severe combined immunodeficiency; OCDs, osteochondral defects; CDs, chondral defects; +, detected; -, undetected; N/A, not announced.

**Table 3:** Implantation conditions of alginate–chondrocyte systems.

Gelation Approach	Gelling Solution	Alginate (wt/vol %)	Cells (10^6^/mL)	Gelling Solution/Alginate	Refs.
adhesion	CaCl_2_ (180 mM)	1.5	120	N/A	[166]
encapsulation	CaCl_2_ (50 mM)	0.75	20–30	N/A	[261]
	CaCl_2_ (102 mM)	1	20	N/A	[262]
	CaCl_2_ (N/A)	2	20	300 μL/500 μL	[259]
	CaCl_2_ (102 mM)	1.2	10	N/A	[82]
	CaCl_2_ (102 mM)	1.2	4	N/A	[211]
	CaCl_2_ (15–100 mM)	0.5–4.0	0/1/5/10	N/A	[138]
	CaSO_4_ (200 mM)	1.8	10	1 mL/2 mL	[260]
	CaSO_4_ (1470 mM)	1	10	0.2 g/1 mL	[258]

Abbreviations: N/A, not announced.

**Table 4:** Implantation methods of alginate–chondrocyte systems.

Method	Preparation	Mixing	Refs.
implantation of two solutions one by one with operation/mixing/gelling	invasive	inadequate	[261]
mixing/gelling implantation with gelation at operation	invasive	adequate	[82,138,166,211,262]
implantation of two solutions one by one with injection via mixing/gelling	not invasive	inadequate	[259]
mixing (Pantaject)-based implantation with injection/gelling	not invasive	inadequate	[260]
mixing (vortex)-based implantation with injection-gelling (low temperature)	not invasive	adequate	[258]

**Table 5:** Biological stimulation of alginate–chondrocyte systems.

Stimulus (ng/mL)		Cells	Alginate, Serum (ds)	Viability, Proliferation	PG Synthesis		PG Deposition		Collagen Deposition	Refs.
					Total	Individual	Total	Individual		
IGF-I	100 (21 ds)	human	-	slight increase	N/A	N/A	N/A	slight increase	N/A	[185]
	100–1000 (28 ds)	human	-	N/A	N/A	increase to decrease	N/A	slight increase	N/A	[266]
	100 (21 ds)	human	-	slight increase	N/A	N/A	slight increase	slight increase	slight increase	[267,268]
	25 (13 ds)	bovine	-, 10% (13 ds)	slight increase	increase	N/A	increase	N/A	N/A	[19]
	100 (7 ds)	human	2.5% (7 ds)	N/A	N/A	N/A	increase	N/A	increase	[269]
	2.5–25 (20–35 ds)	bovine	10% (20–35 ds)	slight decrease	N/A	N/A	increase	N/A	increase	[270]
	100 (14 ds)	human	10% (14 ds)	increase	N/A	increase	N/A	N/A	N/A	[271]
TGF-β	10 (7–28 ds)	rabbit	10% (7–28 ds)	slight increase	N/A	N/A	N/A	increase	N/A	[84]
	25 (13 ds)	bovine	-,10% (13 ds)	slight increase	increase	N/A	increase	N/A	N/A	[19]
	2.5–25 (20–35 ds)	bovine	10% (20–35 ds)	slight decrease	N/A	N/A	decrease	N/A	decrease	[270]
FGF-2	1–100 (21 ds)	human	-	increase	N/A	slight decrease	N/A	decrease	N/A	[268]
	2.5–25 (20–35 ds)	bovine	10% (20–35 ds)	slight increase	N/A	N/A	increase	N/A	increase to decrease	[270]
OP-1	100 (21 ds)	human	-	increase	N/A	slight increase	increase	increase	increase	[185,266,267,268]
	50 (7 ds)	human	10% (7 ds)	slight increase	increase	N/A	N/A	N/A	N/A	[272]
	100 (14 ds)	bovine	5% (14 ds)	N/A	N/A	slight increase	N/A	N/A	N/A	[193]
	100 (14 ds)	bovine	10% (14 ds)	increase	N/A	N/A	increase	increase	increase	[273]
	200–1000 (21 ds)	human	-, 10% (21 ds)	N/A	N/A	N/A	increase	N/A	N/A	[274]
	100 (14 ds)	human	10% (14 ds)	increase	N/A	increase	N/A	N/A	N/A	[271]
BMP-2	100 (14 ds)	human	10% (14 ds)	N/A	N/A	increase	N/A	N/A	N/A	[80]

Abbreviations: IGF-I, insulin-like growth factor I; TGF-β, transforming growth factor beta; FGF-2, basic fibroblast growth factor; OP-1, osteogenic protein 1; BMP-2, bone morphogenetic protein 2; -, free of serum; N/A, not announced; PG, proteoglycans.

**Table 6:** Physicochemical stimulation of alginate–chondrocyte systems.

Stimulus		Cells	Alginate, Serum (ds)	Proliferation	PG Synthesis		PG Deposition		Collagen Synthesis	Collagen Deposition	Refs.
					Total	Individual	Total	Individual			
tension	0–40% compression, 0 Hz, 22 h	bovine	10% (21 ds)	N/A	decrease	N/A	N/A	N/A	N/A	N/A	[20]
	20% sin. strain, 0.5 Hz, 22 h	bovine	10% (21 ds)	N/A	increase	N/A	N/A	N/A	N/A	N/A	[20]
	15–50% sin. strain, 1 Hz, 1–3 h	human dedifferentiated	- (14 ds)	N/A	N/A	increase	N/A	N/A	increase	N/A	[282]
shearing force, perfusion	0.025 mL/h, 9 ds	bovine	-	slight decrease	N/A	N/A	N/A	increase	N/A	increase	[283]
	180 mL/h, 7–14 ds	bovine dedifferentiated	10%	increase	N/A	increase	N/A	N/A	decrease to increase	N/A	[284]
	6 mL/h, 16 ds	human dedifferentiated	10%	N/A	N/A	slight effect	N/A	decrease	increase	N/A	[281]
hydrostatic pressure	1.2–2.4 MPa, 0 Hz, 4 h	goat	10%	N/A	N/A	N/A	N/A	decrease	N/A	decrease	[285]
	1.2–2.4 MPa, 0.66 Hz, 4 h	goat	10%	N/A	N/A	N/A	N/A	increase	N/A	decrease	[285]
	10 MPa, 0 Hz, 12 h	rabbit	10%	N/A	N/A	increase	N/A	N/A	increase	N/A	[279]
	50 MPa, 0 Hz, 12 h	rabbit	10%	N/A	N/A	decrease	N/A	N/A	decrease	N/A	[279]
oxygen tension	0–20%, 7 ds	bovine	10%	slight effect	N/A	slight effect	N/A	N/A	N/A	N/A	[286]
	1–21%, 14 ds	equine	-	slight effect	N/A	N/A	N/A	N/A	N/A	N/A	[287]
	5%, 7–28 ds	bovine dedifferentiated	10%	N/A	N/A	increase	N/A	N/A	increase	N/A	[203]
	5%, 21 ds	bovine dedifferentiated	10%	N/A	N/A	N/A	increase	N/A	N/A	increase	[219]
	5%, 21 ds	bovine dedifferentiated	10%	slight effect	N/A	increase	N/A	N/A	N/A	increase	[36]
	5%, 28 ds	human dedifferentiated	10%	increase	N/A	increase	N/A	increase	increase	N/A	[288]
	5–21%, 14 ds	human dedifferentiated	10%	slight effect	N/A	N/A	N/A	N/A	N/A	slight effect	[289]
osmotic pressure	280 mOsm, 12 ds	bovine	10%	increase	N/A	N/A	N/A	decrease	N/A	slight effect	[290]
	280–550 mOsm, 5 ds	bovine	10%	slight effect	N/A	N/A	N/A	slight effect	N/A	slight effect	[290]
	550 mOsm, 12 ds	bovine	10%	slight effect	N/A	N/A	N/A	slight effect	N/A	slight effect	[290]
	270–570 mOsm, 2–6 ds	bovine	6%	N/A	N/A	decrease	decrease	N/A	N/A	N/A	[291]
	270 mOsm, 2 ds	bovine	-	N/A	N/A	decrease	N/A	N/A	N/A	N/A	[292]
	550 mOsm, 2 ds	bovine	-	N/A	N/A	increase	N/A	N/A	N/A	N/A	[292]
ultrasounds	2 mW/cm^2^, 1.5 MHz, 20 min	chick	10%	slight effect	N/A	decrease	slight effect	N/A	decrease to increase	increase	[293]
	30 mW/cm^2^, 1.5 MHz, 20 min	chick	10%	decrease	N/A	slight effect	slight effect	N/A	increase	slight effect	[293]
	100 mW/cm^2^, 1.5 MHz, 20 min	human dedifferentiated	10%	slight effect	increase	N/A	increase	N/A	increase	slight effect	[294]

Abbreviations: sin. strain, sinusoidal strain; -, free of serum; N/A, not announced; PG, proteoglycans.

**Table 7:** Alginate–progenitor cell systems in vitro (differentiation after encapsulation) and in vivo (encapsulation after differentiation).

System	Cells	Differentiation After Encapsulation	Differentiation Before Encapsulation, Implantation	Proliferation	PG Synthesis	PG Deposition		Collagen Synthesis	Collagen Deposition		Refs.
					Individual	Total	Individual		Total	Individual	
in vitro	rabbit (BM)	2 ds	-	N/A	detected	N/A	N/A	detected	N/A	N/A	[8]
	human (BM)	24 ds TGF-β3	-	decrease	increase	increase	N/A	increase	increase	N/A	[319]
	human (SCS)	14–28 ds TGF-β3	-	N/A	increase	detected	N/A	increase	detected	N/A	[320]
	human (BM)	19 ds TGF-β3	-	N/A	N/A	N/A	increase to decrease	N/A	N/A	increase to decrease	[318]
	bovine (BM)	6 ds TGF-β1	-	N/A	detected	detected	N/A	detected	detected	N/A	[321]
	human (BM)	21 ds TGF-β1/BMP-2	-	N/A	increase	increase	N/A	increase	detected	N/A	[322]
	rabbit (BM)	14 ds TGF-β3/LIUS	-	N/A	detected	detected	N/A	detected	detected	N/A	[323]
	human (A)	21 ds TGF-β3/PRC	-	N/A	detected	detected	N/A	detected	detected	N/A	[325]
	human (BM)	24 ds PRC	-	increase to decrease	increase	increase	increase to decrease	increase	increase	N/A	[324]
	mouse (iPSCs)	7–28 ds BMP-2	-	N/A	detected	N/A	N/A	detected	N/A	N/A	[313]
	human (iPSCs)	21 ds TGF-β3	-	N/A	N/A	N/A	detected	N/A	N/A	N/A	[314]
	murine (iPSCs)	21 ds FGF-2	-	decrease	increase	increase	N/A	increase	increase	N/A	[327]
	human (iPSCs)	21 ds TGF-β1/BMP-2	-	increase	increase	N/A	detected	detected	N/A	N/A	[316]
in vivo	human (A)	-	s.c. (dorsal) nude mice, 20 wk	N/A	detected	increase	N/A	detected	increase	N/A	[328]
	human (A)	-	s.c. (dorsal) nude mice, 12 wk	N/A	N/A	detected	N/A	N/A	detected	N/A	[329]
	rat (BM)	-	s.c. (dorsal) nude mice, 8 wk	N/A	N/A	detected	N/A	N/A	N/A	N/A	[330]
	mouse (iPSCs)	-	s.c. (dorsal) nude mice, 7–28 ds	N/A	detected	detected	N/A	detected	N/A	N/A	[313]

Abbreviations: BM, bone marrow; SCS, subchondral cortico-spongiosa; A, adipose tissue; iPSCs, induced pluripotent stem cells; TGF-β, transforming growth factor beta (10 ng/mL); BMP-2, bone morphogenetic protein 2 (500 ng/mL); LIUS, low-intensity ultrasounds (10 Min/12 H, 200 mW/cm^2^, 1 MHz); PRC, platelet-rich concentrate (15%); FGF-2, basic fibroblast growth factor; -, not applicable; s.c., subcutaneous; N/A, not announced; PG, proteoglycans.

**Table 8:** Alginate combined with gene therapy.

Approach	Vector	Gene	Cells	Evaluation	Proliferation	PG Deposition	Collagen Deposition	Refs.
genetically modified cells in alginate	nonviral	IGF-I	rabbit chondrocytes	in vitro, 36 ds	increase	N/A	N/A	[145]
			rabbit chondrocytes	in vivo (OCD implantation), 14 wk	N/A	increase	slight effect	[145]
		FGF-2	rabbit chondrocytes	in vitro, 29 ds	increase	slight decrease	N/A	[68]
			rabbit chondrocytes	in vivo (OCD implantation), 3/14 wk	N/A	increase	increase	[68]
	adenoviral	IGF-I	ovine ASCs	in vitro, 28 ds	N/A	detected	detected	[346]
		TGF-β1	rat MSCs	in vitro, 10 ds	N/A	detected	detected	[347]
		TGF-β2	human ASCs	in vivo (s.c. implantation), 4/12 wk	N/A	detected	detected	[146]
	lentiviral	TGF-β1	rat MSCs	in vivo (CD implantation), 4/8 wk	N/A	detected	detected	[348]
gene transfer vectorsin alginate	rAAV	*lacZ*	human MSCs	in vitro, 21 ds	N/A	not altered	not altered	[341]
		IGF-I	-	in vivo (CD implantation), 1 yr	increase	increase	increase	[341]

Abbreviations: rAAV, recombinant adeno-associated virus (vector); IGF-I, insulin-like growth factor I; FGF-2, basic fibroblast growth factor; TGF-β, transforming growth factor beta; *lacZ*, Escherichia coli β-galactosidase gene; ASCs, adipose-derived stromal cells (progenitors); MSCs, mesenchymal stromal cells (progenitors); -, not applicable; OCD, osteochondral defect; s.c., subcutaneous; CD, chondral defect; N/A, not announced; PG, proteoglycans.
